# Non-ECG-triggered perfusion imaging with integrated T1 mapping for quantifying myocardial blood flow

**DOI:** 10.1186/1532-429X-16-S1-P202

**Published:** 2014-01-16

**Authors:** David Chen, Behzad Sharif, Janet Wei, Reza Arsanjani, Xiaoming Bi, C Noel Bairey Merz, Daniel S Berman, Debiao Li

**Affiliations:** 1 Biomedical Engineering, Northwestern University, Evanston, Illinois, USA; 2 BIRI, Cedars-Sinai Medical Center, Los Angeles, California, USA; 3 S. Mark Taper Foundation Imaging Center, Cedars Sinai Medical Center, Los Angeles, California, USA; 4 Siemens Medical Solutions, Los Angeles, California, USA; 5 Women's Heart Center, Cedars-Sinai Heart Institute, Los Angeles, California, USA; 6 Bioengineering, University of California, Los Angeles, Los Angeles, California, USA

## Background

The dual bolus method is conventionally used for quantification of myocardial blood flow (MBF). The method necessitates an additional scan for each first-pass measurement, which increases total scan time and is sensitive to change in heart rates between the two scans. A single scan method to measure the arterial input function (AIF) using highly constrained back projection (HYPR) reconstruction for integrated T1 mapping was previously developed [1]. Mistriggering due to arrhythmias, changing heart rates, and poor electrocardiogram (ECG) signal may cause motion artifacts in HYPR reconstructed images because data is shared across multiple cardiac cycles. We propose using a non-ECG-triggered acquisition with cardiac motion self-triggering for integrated T1 mapping derived AIF for MBF quantification.

## Methods

210 measurements were acquired continuously with no ECG triggering. 60 projections were acquired immediately following a saturation recovery (SR) magnetization preparation with a golden angle trajectory. Cardiac motion self-triggering images were produced from 60 projections using non-Cartesian SENSE. A sliding window was used achieve a temporal resolution of 43 ms. The mean signal intensity in a region of interest drawn over the heart was measured. Peaks corresponding to increased ventricular blood pool volume in diastole were used for triggering. T1 maps were produced by measuring longitudinal magnetization recovery following a saturation recovery preparation [1]. Contrast agent concentration in the blood pool were found using T1 values. MBF was found using linear time invariant model-independent deconvolution. Six healthy volunteers underwent rest perfusion MRI studies on a Siemens 3T Verio. First, a dual bolus protocol was performed using a conventional clinical Cartesian sequence [2]. 10 min following the first perfusion scan, a second first pass perfusion experiment was performed using the proposed method. A two sided Student t-test was performed to MBF at a p = 0.05 significance level.

## Results

Mean MBF found from the dual bolus and proposed non-ECG triggered, integrated T1 mapping acquisitions were 0.81 ± 0.29 ml/min/g and 0.93 ± 0.21 ml/min/g. There was no significant difference (p = 0.43) between MBF found using dual bolus and proposed acquisitions.

## Conclusions

Non-ECG-triggered perfusion MR with fast T1 mapping for integrated AIF measurement produces similar MBF as gold standard dual bolus method. Fast T1 mapping alleviates the need for a separate scan specifically used for the acquisition of the AIF. Non-ECG-triggered imaging may improve robustness to arrhythmias and changing heart rates. This method may improve clinical feasibility of quantitative myocardial perfusion imaging.

## Funding

NIH Grant T32 EB51705. NIH Grant RO1 EB002623. AHA Postdoctoral Fellowship Award 1POST7390063.

**Figure 1:**
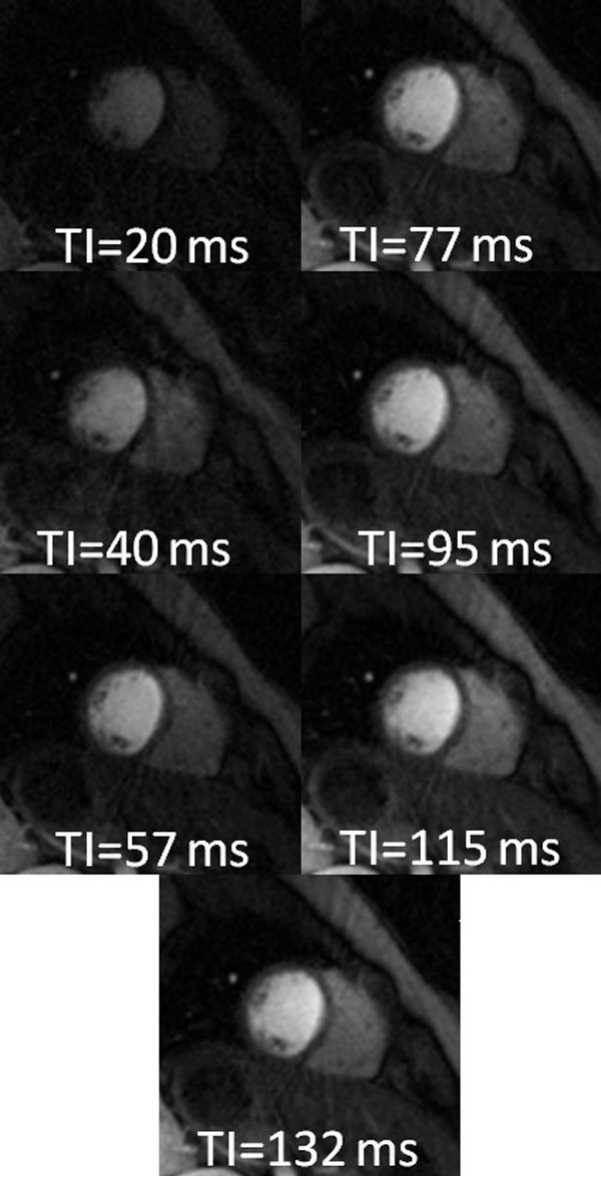
**Non-ECG triggered images of each TI during a single cardiac cycle**. Images were created from 15 projections and used for T1 mapping.

**Figure 2:**
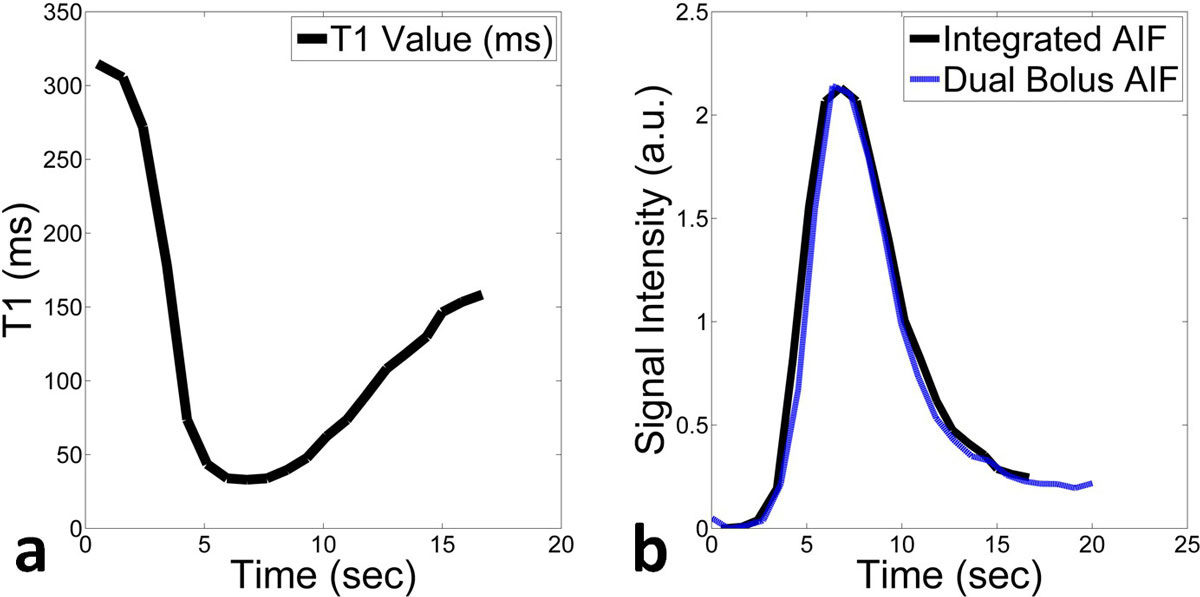
**a) Change in T1 values in the left ventricular blood pool**. b) Comparison of the arterial input function derived from non-ECG-triggered integrated T1 mapping protocol and the dual bolus protocol.
